# 4,6-Diferrocenyl-5-(morpholin-4-yl)-1,2,3-triazine

**DOI:** 10.1107/S2414314623006168

**Published:** 2023-08-01

**Authors:** Carla Aguilar-Lugo, Marcos Flores-Alamo, Jessica J. Sánchez García, Elena I. Klimova

**Affiliations:** a Facultad de Química, Universidad Nacional Autónoma de México, Ciudad Universitaria, Ciudad de México, 04510, Mexico; Benemérita Universidad Autónoma de Puebla, México

**Keywords:** crystal structure, morpholine, ferrocen­yl, triazine

## Abstract

The structure of the title Fe complex, [Fe(C_5_H_5_)_2_(C_17_H_16_N_4_O)], was determined at 130 K, and has ortho­rhom­bic (*Pna*2_1_) symmetry. It is of inter­est with respect to the class of triazine heterocyclic compounds: the triazine ring is substituted by two ferrocenyl and one morpholine groups. The crystal structure features C—H⋯O and C—H⋯N non-classical hydrogen bonds.

## Abstract 2

The title complex crystallizes with two independent mol­ecules in the asymmetric unit of the ortho­rhom­bic unit cell. The crystal structure features C—H⋯O and C—H⋯N non-classical hydrogen bonds.

## Graphical abstract

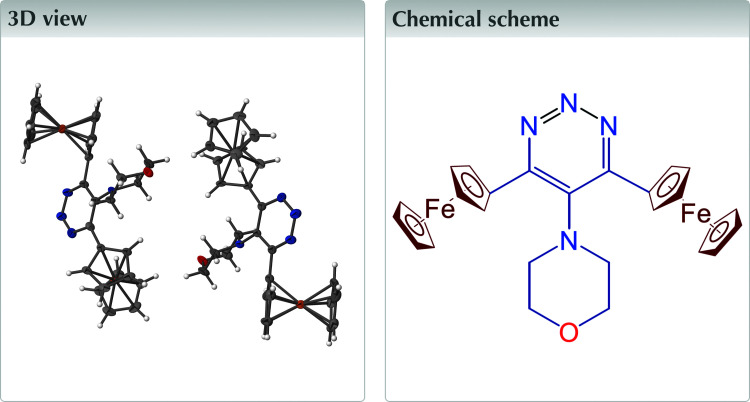

## Structure description

1,2,3-Triazines form an inter­esting class of heterocyclic compounds. Various synthetic analogues of 1,2,3-triazines have been prepared and evaluated for many pharmacological activities, for example: anti­bacterial and antiviral (Migawa *et al.*, 2005 ▸), anti­biotic (Rosowsky *et al.*, 1992 ▸), anti­cancer (Garuti *et al.*, 1998 ▸), anti­microbial (Saravanan *et al.*, 2010 ▸), anti­fungal (Hunt *et al.*, 2007 ▸), anti­protozoal (Quintela *et al.*, 2003 ▸), nematocidal (Kiuchi *et al.*, 1992 ▸), anti­histaminic (Quintela *et al.*, 1998 ▸), analgesic, anti-inflammatory and anti­arthritic activities (Viswanatha *et al.*, 2011 ▸). On the other hand, ferrocene [Fe(C_5_H_5_)_2_] has a stable sandwich structure; the incorporation of ferrocenyl into biological mol­ecules offers the potential to develop better and more efficient therapeutic drugs. Thus, substituting the 1,2,3-triazine heterocycle with two ferrocenyl groups can lead to mol­ecules with pharmacological activity, useful for the development of future drugs.

**Figure 1:**
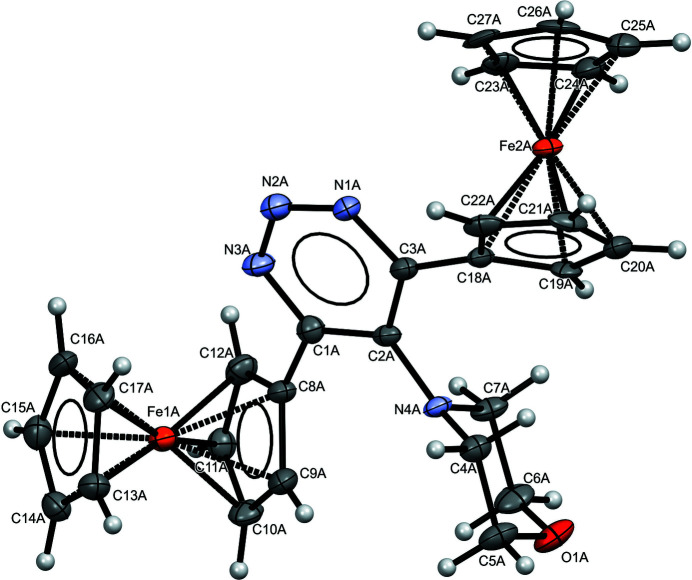
*ORTEP* diagram of the title compound. One mol­ecule of the asymmetric unit is displayed. Displacement ellipsoids for non-H atoms are drawn at the 50% probability level.

**Figure 2:**
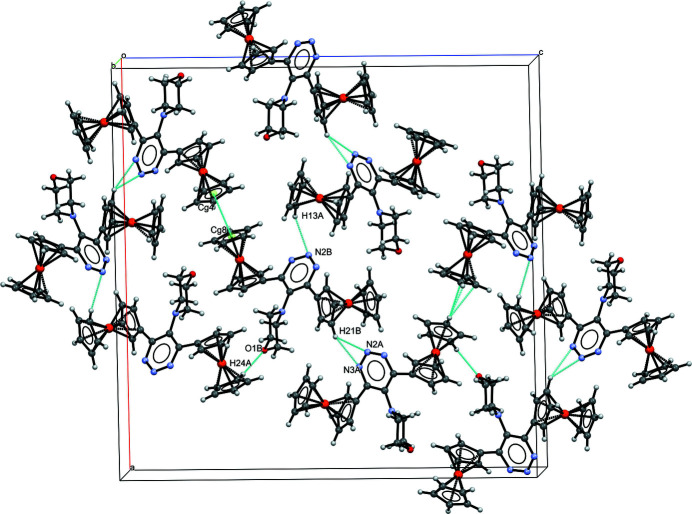
Part of the crystal structure of the title compound, viewed along [010] and showing inter­molecular contacts of the type C—H⋯O, N—H⋯O and π–π (dashed blue lines).

The title compound (Fig. 1 ▸), crystallizes with two independent mol­ecules of 5-(morpholino)-4,6-diferrocenyl-1,2,3-triazine in the asymmetric unit. Each mol­ecule (labelled *A* and *B*) is constituted by a pair of ferrocenyl complexes bonded to the triazine ring; moreover, C2*A* and C2*B* are bonded to six-membered morpholine groups (Fig. 2 ▸). The morpholine ring assumes a conformation very close to a chair conformation, with puckering parameters for mol­ecule *A*: *q* = 0.563 (9) Å, θ = 4.8 (8)° and φ = 27 (11)° if the calculation starts from O1*A* to C6*A* and proceeds in a clockwise direction. For mol­ecule *B*, puckering parameters are; *q* = 0.569 (8) Å, θ = 174.7 (8)° and φ = 237 (8)° if the calculation starts from O1*B* to C6*B* and proceeds in a clockwise direction.

In the crystal, mol­ecules *A* and *B* are linked *via* C—H⋯O and C—H⋯N non-classical hydrogen bonds (Table 1 ▸); additionally there are π–π inter­actions. The inter­molecular C21*B*—H21*B*⋯N2*A* (2.56 Å) and C21*B*—H21*B*⋯N3*A* (2.52 Å) inter­actions form a 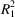 (3) ring motif, while C24*A*—H24*A*⋯O1*B* has an inter­action distance of 2.51 Å; on the other hand, weak π–π inter­actions involve the five-membered cyclo­penta­dienyl rings, *Cg*(C23*A*–C27*A*)⋯*Cg*(C13*B*–C17*B*)( 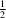 + *x*, 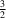 − *y*, *z*) = 4.332 (5) Å with slippage = 0.111 Å. All of these inter­molecular inter­actions form slabs lying parallel to the *ac* plane in the crystal (Fig. 2 ▸).

## Synthesis and crystallization

**Figure 3:**
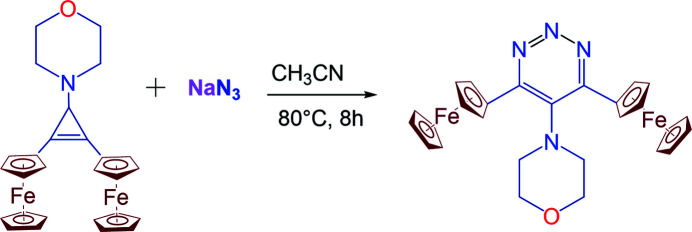
Synthesis of the title compound.

Sodium azide (1.3 g, 20 mmol) was added to a solution of 1-morpholino-2,3-diferrocenyl­cyclo­propenylium tetra­fluoro­borate (10 mmol) in aceto­nitrile (100 ml), and the mixture was stirred in a dry inert atmosphere under reflux for 8 h (Fig. 3 ▸). The solvents were removed *in vacuo*, and the residues were chromatographed on alumina (eluent: hexane-di­chloro­methane, 4:1). Crystals of the title compound suitable for single-crystal diffraction analysis were obtained by slow evaporation of a saturated di­chloro­methane/hexane (ratio 1:1 *v*/*v*) solution. Yield: 65%, red crystals, m.p. 498–500 K. ^1^H-NMR (400 MHz, CDCl_3_) δ: 2.83 (4 H, *m*, CH_2_), 3.64 (4 H, *m*, CH_2_), 4.22 (10 H, *s*, 2 C_5_H_5_), 4.47 (4 H, *m*, C_5_H_4_), 4.94 (4 H, *m*, C_5_H_4_) p.p.m. ^13^C-NMR (75 MHz, CDCl_3_) δ: 49.87 (2 CH_2_), 66.54 (2 CH_2_), 81.79 (2 C_*ipso*_ Fc), 70.52 (2 C_5_H_5_), 69.81, 71.34 (2 C_5_H_4_), 138.64, 156.17 (2 C) p.p.m. MS: *m*/*z* 534 [*M*]^+^. Analysis calculated for C_27_H_26_Fe_2_N_4_O: C, 60.70, H, 4.91, N, 10.48%; found C, 60.85, H, 5.01, N, 10.39%.

## Refinement

Crystal data, data collection and structure refinement details are summarized in Table 2 ▸. The structure was refined considering the crystal as a racemic twin, and the batch scale factor converged towards 0.55 (3) (Sheldrick, 2015*b* ▸).

## Supplementary Material

Crystal structure: contains datablock(s) global, I. DOI: 10.1107/S2414314623006168/bh4075sup1.cif

Structure factors: contains datablock(s) I. DOI: 10.1107/S2414314623006168/bh4075Isup2.hkl

CCDC reference: 2281449

Additional supporting information:  crystallographic information; 3D view; checkCIF report

## Figures and Tables

**Table 1:** Hydrogen-bond geometry (Å, °)

*D*—H⋯*A*	*D*—H	H⋯*A*	*D*⋯*A*	*D*—H⋯*A*
C21*B*—H21*B*⋯N2*A* ^i^	0.95	2.56	3.300 (12)	135
C21*B*—H21*B*⋯N3*A* ^i^	0.95	2.52	3.415 (10)	156
C24*A*—H24*A*⋯O1*B*	0.95	2.51	3.401 (10)	156

Symmetry code: (i) 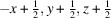 .

**Table 2:** Experimental details

Crystal data	
Chemical formula	[Fe(C_5_H_5_)_2_(C_17_H_16_N_4_O)]
*M*_r_	534.22
Crystal system, space group	Orthorhombic, *P* *n* *a*2_1_
Temperature (K)	130
*a*, *b*, *c* (Å)	27.0967 (19), 5.9846 (4), 27.448 (2)
*V* (Å^3^)	4451.1 (6)
*Z*	8
Radiation type	Mo *K*α
μ (mm^−1^)	1.33
Crystal size (mm)	0.52 × 0.20 × 0.05

Data collection	
Diffractometer	Xcalibur, Atlas, Gemini
Absorption correction	Analytical (*CrysAlis RED*; Agilent, 2013 ▸)
*T*_min_, *T*_max_	0.678, 0.925
No. of measured, independent and observed [*I* > 2σ(*I*)] reflections	16624, 8379, 6445
*R*_int_	0.060
(sin θ/λ)_max_ (Å^−1^)	0.695

Refinement	
*R*[*F* ^2^ > 2σ(*F* ^2^)], *wR*(*F* ^2^), *S*	0.053, 0.123, 1.04
No. of reflections	8379
No. of parameters	602
No. of restraints	1
H-atom treatment	H-atom parameters constrained
Δρ_max_, Δρ_min_ (e Å^−3^)	0.59, −1.18
Absolute structure	Refined as an inversion twin
Absolute structure parameter	0.55 (3)

Computer programs: *CrysAlis PRO* (Agilent, 2013 ▸), *CrysAlis RED* Agilent, 2013 ▸, *SHELXT2018/2* (Sheldrick, 2015*a* ▸), *SHELXL2018/3* (Sheldrick, 2015*b* ▸), *Mercury* (Macrae *et al.*, 2020 ▸) and *publCIF* (Westrip, 2010 ▸).

## full crystallographic data

### Crystal data

**Table:**

[Fe(C_5_H_5_)_2_(C_17_H_16_N_4_O)]	*F*(000) = 2208
*M**_r_* = 534.22	*D*_x_ = 1.594 Mg m^−^^3^
Orthorhombic, *P**n**a*2_1_	Mo *K*α radiation, λ = 0.71073 Å
Hall symbol: P 2c -2n	Cell parameters from 2964 reflections
*a* = 27.0967 (19) Å	θ = 4.1–27.3°
*b* = 5.9846 (4) Å	µ = 1.33 mm^−^^1^
*c* = 27.448 (2) Å	*T* = 130 K
*V* = 4451.1 (6) Å^3^	Plate, intense red
*Z* = 8	0.52 × 0.20 × 0.05 mm

### Data collection

**Table:**

Xcalibur, Atlas, Gemini diffractometer	8379 independent reflections
Graphite monochromator	6445 reflections with *I* > 2σ(*I*)
Detector resolution: 10.4685 pixels mm^-1^	*R*_int_ = 0.060
ω scans	θ_max_ = 29.6°, θ_min_ = 3.5°
Absorption correction: analytical (*CrysAlis RED*; Agilent, 2013)	*h* = −20→34
*T*_min_ = 0.678, *T*_max_ = 0.925	*k* = −7→8
16624 measured reflections	*l* = −37→29

### Refinement

**Table:**

Refinement on *F*^2^	Hydrogen site location: inferred from neighbouring sites
Least-squares matrix: full	H-atom parameters constrained
*R*[*F*^2^ > 2σ(*F*^2^)] = 0.053	*w* = 1/[σ^2^(*F*_o_^2^) + (0.0351*P*)^2^ + 7.4132*P*] where *P* = (*F*_o_^2^ + 2*F*_c_^2^)/3
*wR*(*F*^2^) = 0.123	(Δ/σ)_max_ < 0.001
*S* = 1.04	Δρ_max_ = 0.59 e Å^−^^3^
8379 reflections	Δρ_min_ = −1.18 e Å^−^^3^
602 parameters	Absolute structure: Refined as an inversion twin
1 restraint	Absolute structure parameter: 0.55 (3)

### Special details

**Table:**

Refinement. Refined as a 2-component inversion twin

### Fractional atomic coordinates and isotropic or equivalent isotropic displacement parameters (Å^2^)

**Table:**

	*x*	*y*	*z*	*U*_iso_*/*U*_eq_	
Fe1A	0.14946 (4)	0.27442 (19)	−0.03751 (4)	0.0216 (3)	
Fe2A	0.25461 (4)	0.95236 (18)	0.21382 (5)	0.0210 (3)	
O1A	0.0288 (2)	0.6092 (10)	0.1568 (2)	0.0326 (15)	
N1A	0.2541 (2)	0.7826 (12)	0.0947 (2)	0.0243 (16)	
N2A	0.2655 (3)	0.6474 (13)	0.0585 (3)	0.0293 (17)	
N3A	0.2340 (2)	0.4966 (12)	0.0419 (3)	0.0270 (16)	
N4A	0.1231 (2)	0.6211 (11)	0.1142 (2)	0.0198 (15)	
C1A	0.1876 (3)	0.4876 (13)	0.0598 (3)	0.0217 (17)	
C2A	0.1726 (3)	0.6295 (13)	0.0982 (3)	0.0183 (16)	
C3A	0.2094 (3)	0.7725 (13)	0.1165 (3)	0.0201 (17)	
C4A	0.0960 (3)	0.8278 (14)	0.1235 (3)	0.0240 (19)	
H4AA	0.104363	0.940944	0.098546	0.029*	
H4AB	0.105009	0.888296	0.155912	0.029*	
C5A	0.0414 (3)	0.7762 (15)	0.1219 (4)	0.030 (2)	
H5AA	0.02238	0.914021	0.12869	0.036*	
H5AB	0.032396	0.723637	0.088857	0.036*	
C6A	0.0538 (3)	0.4081 (15)	0.1456 (4)	0.032 (2)	
H6AA	0.043649	0.356194	0.112888	0.039*	
H6AB	0.044234	0.291874	0.169518	0.039*	
C7A	0.1095 (3)	0.4389 (14)	0.1467 (3)	0.0252 (19)	
H7AA	0.12034	0.473436	0.180345	0.03*	
H7AB	0.125923	0.299308	0.136237	0.03*	
C8A	0.1551 (3)	0.3207 (14)	0.0363 (3)	0.0209 (18)	
C9A	0.1047 (3)	0.3429 (15)	0.0206 (3)	0.0282 (19)	
H9A	0.084662	0.471932	0.024163	0.034*	
C10A	0.0904 (3)	0.1388 (14)	−0.0011 (3)	0.0278 (19)	
H10A	0.058811	0.10658	−0.01447	0.033*	
C11A	0.1309 (3)	−0.0084 (13)	0.0005 (3)	0.0254 (19)	
H11A	0.131055	−0.157796	−0.011219	0.03*	
C12A	0.1713 (3)	0.1027 (13)	0.0224 (3)	0.0271 (19)	
H12A	0.203465	0.04305	0.026931	0.032*	
C13A	0.1390 (3)	0.5181 (15)	−0.0897 (3)	0.0272 (19)	
H13A	0.115856	0.637006	−0.088652	0.033*	
C14A	0.1312 (3)	0.3035 (15)	−0.1096 (3)	0.028 (2)	
H14A	0.101409	0.254012	−0.124344	0.033*	
C15A	0.1742 (3)	0.1745 (16)	−0.1043 (3)	0.030 (2)	
H15A	0.178367	0.023806	−0.114375	0.036*	
C16A	0.2105 (3)	0.3116 (17)	−0.0809 (3)	0.032 (2)	
H16A	0.243352	0.26903	−0.073186	0.038*	
C17A	0.1885 (3)	0.5228 (14)	−0.0714 (3)	0.028 (2)	
H17A	0.20388	0.64572	−0.055648	0.034*	
C18A	0.2044 (3)	0.9224 (13)	0.1580 (3)	0.0191 (17)	
C19A	0.1812 (3)	0.8843 (14)	0.2044 (3)	0.0238 (19)	
H19A	0.164532	0.751976	0.214149	0.029*	
C20A	0.1875 (3)	1.0803 (14)	0.2329 (3)	0.0251 (19)	
H20A	0.175742	1.10058	0.265183	0.03*	
C21A	0.2139 (3)	1.2386 (13)	0.2060 (3)	0.027 (2)	
H21A	0.222689	1.384315	0.216601	0.032*	
C22A	0.2252 (3)	1.1428 (14)	0.1600 (3)	0.0255 (19)	
H22A	0.243497	1.212901	0.134693	0.031*	
C23A	0.3064 (3)	0.7036 (14)	0.2078 (3)	0.027 (2)	
H23A	0.304822	0.578863	0.186469	0.032*	
C24A	0.2880 (3)	0.7119 (15)	0.2563 (3)	0.028 (2)	
H24A	0.271793	0.593843	0.273014	0.033*	
C25A	0.2981 (3)	0.9247 (15)	0.2752 (3)	0.031 (2)	
H25A	0.289896	0.975314	0.307003	0.037*	
C26A	0.3222 (3)	1.0502 (16)	0.2392 (3)	0.031 (2)	
H26A	0.333167	1.200262	0.242403	0.037*	
C27A	0.3274 (3)	0.9133 (17)	0.1970 (3)	0.033 (2)	
H27A	0.342272	0.955672	0.16711	0.04*	
Fe1B	−0.01971 (4)	0.07528 (19)	0.28376 (4)	0.0221 (3)	
Fe2B	0.10746 (4)	0.77639 (19)	0.52417 (4)	0.0228 (3)	
O1B	0.2098 (2)	0.4205 (9)	0.3295 (2)	0.0255 (13)	
N1B	−0.0128 (2)	0.2531 (13)	0.4044 (3)	0.0271 (16)	
N2B	−0.0209 (2)	0.3894 (13)	0.4413 (3)	0.0298 (18)	
N3B	0.0118 (2)	0.5426 (11)	0.4549 (3)	0.0258 (16)	
N4B	0.1163 (2)	0.4145 (10)	0.3742 (2)	0.0182 (14)	
C1B	0.0562 (3)	0.5520 (14)	0.4325 (3)	0.0209 (10)	
C2B	0.0678 (3)	0.4078 (13)	0.3941 (3)	0.0209 (10)	
C3B	0.0300 (3)	0.2656 (13)	0.3783 (3)	0.0222 (18)	
C4B	0.1425 (3)	0.2039 (13)	0.3644 (3)	0.0192 (17)	
H4BA	0.132923	0.143948	0.332156	0.023*	
H4BB	0.134292	0.091376	0.389563	0.023*	
C5B	0.1979 (3)	0.2558 (13)	0.3654 (3)	0.0219 (18)	
H5BA	0.207263	0.311268	0.39807	0.026*	
H5BB	0.216824	0.117456	0.35888	0.026*	
C6B	0.1846 (3)	0.6241 (14)	0.3398 (3)	0.0267 (19)	
H6BA	0.1933	0.736431	0.314769	0.032*	
H6BB	0.195765	0.681772	0.371804	0.032*	
C7B	0.1289 (3)	0.5939 (13)	0.3408 (3)	0.0214 (17)	
H7BA	0.112947	0.734418	0.351446	0.026*	
H7BB	0.116849	0.557518	0.307701	0.026*	
C8B	0.0325 (3)	0.1105 (14)	0.3374 (3)	0.0207 (17)	
C9B	0.0115 (3)	−0.1096 (13)	0.3374 (3)	0.0232 (18)	
H9B	−0.005802	−0.177343	0.363607	0.028*	
C10B	0.0211 (3)	−0.2081 (13)	0.2914 (3)	0.0262 (19)	
H10B	0.011845	−0.354932	0.282004	0.031*	
C11B	0.0465 (3)	−0.0551 (15)	0.2622 (3)	0.029 (2)	
H11B	0.056835	−0.078036	0.229503	0.034*	
C12B	0.0539 (3)	0.1434 (13)	0.2908 (3)	0.0209 (10)	
H12B	0.070445	0.274803	0.280236	0.025*	
C13B	−0.0689 (3)	0.3330 (14)	0.2876 (4)	0.029 (2)	
H13B	−0.065349	0.46345	0.307053	0.035*	
C14B	−0.0914 (3)	0.1312 (15)	0.3027 (3)	0.027 (2)	
H14B	−0.105514	0.10226	0.333729	0.033*	
C15B	−0.0891 (3)	−0.0168 (16)	0.2639 (4)	0.038 (2)	
H15B	−0.101375	−0.165396	0.263929	0.046*	
C16B	−0.0654 (3)	0.0894 (16)	0.2243 (3)	0.036 (2)	
H16B	−0.058988	0.025676	0.193245	0.044*	
C17B	−0.0528 (3)	0.3091 (15)	0.2395 (3)	0.027 (2)	
H17B	−0.036496	0.419328	0.220539	0.032*	
C18B	0.0901 (3)	0.7245 (14)	0.4520 (3)	0.0215 (18)	
C19B	0.0750 (3)	0.9403 (13)	0.4685 (3)	0.0251 (19)	
H19B	0.042118	0.995539	0.468768	0.03*	
C20B	0.1177 (3)	1.0583 (15)	0.4843 (3)	0.029 (2)	
H20B	0.118361	1.207343	0.4962	0.035*	
C21B	0.1586 (3)	0.9158 (15)	0.4792 (3)	0.029 (2)	
H21B	0.191746	0.952837	0.487196	0.034*	
C22B	0.1425 (3)	0.7077 (15)	0.4602 (3)	0.0279 (19)	
H22B	0.162601	0.580713	0.453929	0.034*	
C23B	0.0751 (3)	0.5369 (15)	0.5659 (3)	0.031 (2)	
H23B	0.056108	0.413747	0.554664	0.038*	
C24B	0.0565 (4)	0.7507 (18)	0.5778 (4)	0.046 (3)	
H24B	0.023083	0.797268	0.575547	0.055*	
C25B	0.0968 (5)	0.8829 (19)	0.5936 (4)	0.054 (3)	
H25B	0.095142	1.034417	0.603786	0.065*	
C26B	0.1394 (4)	0.7508 (16)	0.5915 (3)	0.040 (3)	
H26B	0.171587	0.797214	0.600622	0.048*	
C27B	0.1266 (3)	0.5374 (16)	0.5736 (3)	0.033 (2)	
H27B	0.148586	0.416574	0.56786	0.04*	

### Atomic displacement parameters (Å^2^)

**Table:**

	*U*^11^	*U*^22^	*U*^33^	*U*^12^	*U*^13^	*U*^23^
Fe1A	0.0158 (6)	0.0257 (6)	0.0233 (6)	0.0010 (5)	−0.0007 (5)	−0.0025 (6)
Fe2A	0.0104 (6)	0.0261 (6)	0.0265 (5)	−0.0010 (5)	−0.0029 (5)	−0.0004 (6)
O1A	0.018 (3)	0.027 (3)	0.053 (4)	−0.002 (3)	0.012 (3)	−0.002 (3)
N1A	0.014 (3)	0.038 (4)	0.021 (3)	0.001 (3)	0.001 (3)	−0.002 (3)
N2A	0.018 (4)	0.041 (4)	0.029 (4)	0.002 (3)	−0.001 (3)	−0.007 (4)
N3A	0.014 (3)	0.036 (4)	0.031 (4)	−0.002 (3)	−0.001 (3)	−0.005 (3)
N4A	0.010 (3)	0.024 (3)	0.025 (4)	0.001 (3)	0.001 (3)	−0.003 (3)
C1A	0.021 (4)	0.022 (4)	0.022 (4)	0.000 (3)	0.000 (3)	0.001 (4)
C2A	0.012 (4)	0.021 (4)	0.022 (4)	0.000 (3)	−0.002 (3)	0.003 (4)
C3A	0.013 (4)	0.026 (4)	0.022 (4)	0.001 (3)	−0.001 (3)	0.001 (4)
C4A	0.018 (4)	0.025 (4)	0.029 (5)	0.001 (3)	0.001 (3)	−0.001 (4)
C5A	0.016 (4)	0.029 (5)	0.045 (5)	0.003 (4)	0.003 (4)	−0.002 (4)
C6A	0.019 (5)	0.030 (5)	0.048 (6)	−0.010 (4)	0.004 (4)	−0.005 (5)
C7A	0.013 (4)	0.022 (4)	0.040 (5)	−0.002 (3)	0.000 (4)	−0.001 (4)
C8A	0.013 (4)	0.027 (4)	0.023 (4)	0.001 (3)	−0.001 (3)	0.001 (4)
C9A	0.016 (4)	0.043 (5)	0.025 (4)	0.000 (4)	0.002 (4)	−0.004 (5)
C10A	0.016 (4)	0.033 (5)	0.035 (5)	−0.007 (4)	0.000 (4)	−0.005 (4)
C11A	0.029 (5)	0.023 (4)	0.024 (4)	0.000 (4)	0.001 (4)	−0.003 (4)
C12A	0.023 (4)	0.027 (4)	0.031 (5)	0.006 (4)	0.003 (4)	0.001 (4)
C13A	0.020 (5)	0.036 (5)	0.026 (5)	0.003 (4)	−0.002 (4)	0.002 (4)
C14A	0.026 (5)	0.036 (5)	0.021 (4)	−0.002 (4)	−0.003 (4)	0.002 (4)
C15A	0.032 (5)	0.037 (5)	0.022 (5)	0.003 (4)	0.004 (4)	−0.001 (4)
C16A	0.016 (4)	0.056 (6)	0.024 (4)	0.008 (4)	0.003 (3)	0.001 (4)
C17A	0.022 (5)	0.034 (5)	0.029 (4)	−0.008 (4)	0.004 (4)	0.003 (4)
C18A	0.011 (4)	0.024 (4)	0.022 (4)	0.002 (3)	−0.002 (3)	−0.007 (4)
C19A	0.010 (4)	0.034 (4)	0.027 (5)	−0.005 (3)	−0.007 (3)	−0.006 (4)
C20A	0.013 (4)	0.037 (5)	0.025 (4)	0.004 (4)	−0.001 (3)	−0.005 (4)
C21A	0.019 (4)	0.024 (4)	0.038 (5)	0.001 (3)	−0.015 (4)	−0.012 (4)
C22A	0.015 (4)	0.023 (4)	0.038 (5)	0.005 (3)	−0.005 (4)	0.007 (4)
C23A	0.015 (4)	0.026 (4)	0.039 (5)	0.012 (3)	−0.005 (4)	−0.003 (4)
C24A	0.012 (4)	0.039 (5)	0.032 (5)	−0.001 (4)	0.000 (3)	0.006 (4)
C25A	0.016 (4)	0.044 (5)	0.032 (5)	0.000 (4)	−0.006 (4)	−0.005 (5)
C26A	0.011 (4)	0.038 (5)	0.044 (5)	0.001 (4)	−0.016 (4)	0.000 (5)
C27A	0.006 (4)	0.054 (6)	0.039 (5)	0.007 (4)	0.003 (4)	0.014 (5)
Fe1B	0.0108 (5)	0.0250 (6)	0.0305 (6)	0.0005 (5)	−0.0054 (5)	−0.0009 (6)
Fe2B	0.0195 (6)	0.0274 (6)	0.0215 (6)	0.0002 (5)	0.0006 (5)	−0.0010 (6)
O1B	0.017 (3)	0.025 (3)	0.034 (3)	−0.002 (2)	0.006 (2)	0.000 (3)
N1B	0.012 (3)	0.039 (4)	0.030 (4)	0.001 (3)	0.005 (3)	0.000 (4)
N2B	0.015 (4)	0.042 (4)	0.033 (4)	−0.004 (3)	0.001 (3)	−0.005 (4)
N3B	0.010 (3)	0.034 (4)	0.033 (4)	−0.001 (3)	0.001 (3)	−0.006 (4)
N4B	0.013 (3)	0.016 (3)	0.025 (3)	−0.001 (3)	0.002 (3)	−0.001 (3)
C1B	0.008 (2)	0.025 (2)	0.029 (3)	0.0004 (18)	−0.0007 (19)	−0.003 (2)
C2B	0.008 (2)	0.025 (2)	0.029 (3)	0.0004 (18)	−0.0007 (19)	−0.003 (2)
C3B	0.011 (4)	0.024 (4)	0.031 (5)	0.001 (3)	0.002 (3)	0.001 (4)
C4B	0.011 (4)	0.019 (4)	0.027 (4)	0.000 (3)	−0.002 (3)	−0.004 (4)
C5B	0.008 (4)	0.026 (4)	0.032 (5)	0.002 (3)	−0.003 (3)	0.002 (4)
C6B	0.018 (4)	0.023 (4)	0.039 (5)	−0.003 (3)	0.007 (4)	−0.002 (4)
C7B	0.018 (4)	0.023 (4)	0.023 (4)	−0.005 (3)	0.002 (3)	0.002 (4)
C8B	0.008 (4)	0.028 (4)	0.026 (4)	0.000 (3)	−0.002 (3)	0.003 (4)
C9B	0.016 (4)	0.020 (4)	0.033 (5)	−0.001 (3)	−0.007 (4)	0.004 (4)
C10B	0.015 (4)	0.024 (4)	0.039 (5)	0.004 (3)	−0.009 (4)	−0.001 (4)
C11B	0.016 (4)	0.037 (5)	0.033 (5)	0.006 (4)	−0.007 (4)	−0.010 (4)
C12B	0.008 (2)	0.025 (2)	0.029 (3)	0.0004 (18)	−0.0007 (19)	−0.003 (2)
C13B	0.018 (4)	0.025 (4)	0.044 (5)	0.004 (3)	−0.007 (4)	−0.005 (5)
C14B	0.012 (4)	0.036 (5)	0.033 (5)	0.004 (4)	−0.002 (3)	0.004 (4)
C15B	0.016 (5)	0.036 (5)	0.064 (7)	−0.004 (4)	−0.014 (4)	0.006 (5)
C16B	0.028 (5)	0.053 (6)	0.028 (5)	0.014 (5)	−0.013 (4)	−0.005 (5)
C17B	0.017 (4)	0.033 (5)	0.030 (5)	0.002 (4)	−0.004 (4)	0.010 (4)
C18B	0.015 (4)	0.031 (4)	0.019 (4)	−0.001 (4)	0.001 (3)	−0.005 (4)
C19B	0.021 (4)	0.032 (4)	0.022 (4)	0.007 (4)	−0.001 (3)	0.000 (4)
C20B	0.035 (5)	0.029 (5)	0.024 (4)	−0.003 (4)	−0.001 (4)	−0.003 (4)
C21B	0.022 (5)	0.041 (5)	0.023 (4)	−0.009 (4)	−0.002 (4)	0.001 (4)
C22B	0.017 (4)	0.041 (5)	0.026 (4)	0.003 (4)	0.002 (4)	−0.002 (5)
C23B	0.033 (5)	0.035 (5)	0.026 (5)	−0.006 (4)	0.007 (4)	0.011 (4)
C24B	0.041 (6)	0.055 (7)	0.042 (6)	0.014 (6)	0.027 (5)	0.005 (5)
C25B	0.094 (10)	0.042 (6)	0.026 (5)	−0.008 (7)	0.008 (6)	−0.003 (5)
C26B	0.050 (7)	0.045 (6)	0.026 (5)	−0.012 (5)	−0.013 (5)	0.007 (5)
C27B	0.028 (5)	0.040 (5)	0.031 (5)	−0.005 (4)	−0.006 (4)	0.010 (4)

### Geometric parameters (Å, º)

**Table:**

Fe1A—C12A	2.026 (9)	Fe1B—C9B	2.026 (8)
Fe1A—C15A	2.041 (8)	Fe1B—C15B	2.032 (9)
Fe1A—C9A	2.044 (9)	Fe1B—C10B	2.036 (8)
Fe1A—C14A	2.048 (8)	Fe1B—C14B	2.038 (8)
Fe1A—C17A	2.048 (8)	Fe1B—C13B	2.042 (8)
Fe1A—C16A	2.050 (9)	Fe1B—C11B	2.043 (8)
Fe1A—C11A	2.051 (8)	Fe1B—C12B	2.046 (7)
Fe1A—C8A	2.051 (8)	Fe1B—C16B	2.049 (8)
Fe1A—C10A	2.055 (8)	Fe1B—C8B	2.052 (8)
Fe1A—C13A	2.063 (8)	Fe1B—C17B	2.058 (8)
Fe2A—C22A	2.030 (8)	Fe2B—C19B	2.019 (8)
Fe2A—C27A	2.038 (8)	Fe2B—C24B	2.023 (9)
Fe2A—C20A	2.041 (8)	Fe2B—C25B	2.030 (10)
Fe2A—C26A	2.045 (8)	Fe2B—C20B	2.030 (9)
Fe2A—C19A	2.048 (8)	Fe2B—C23B	2.034 (8)
Fe2A—C21A	2.049 (8)	Fe2B—C21B	2.036 (9)
Fe2A—C23A	2.053 (8)	Fe2B—C27B	2.039 (9)
Fe2A—C18A	2.057 (8)	Fe2B—C22B	2.039 (9)
Fe2A—C24A	2.062 (9)	Fe2B—C26B	2.046 (9)
Fe2A—C25A	2.062 (8)	Fe2B—C18B	2.059 (8)
O1A—C6A	1.414 (10)	O1B—C6B	1.426 (10)
O1A—C5A	1.426 (11)	O1B—C5B	1.430 (10)
N1A—N2A	1.319 (10)	N1B—N2B	1.320 (10)
N1A—C3A	1.354 (10)	N1B—C3B	1.366 (10)
N2A—N3A	1.323 (10)	N2B—N3B	1.328 (9)
N3A—C1A	1.350 (10)	N3B—C1B	1.353 (10)
N4A—C2A	1.414 (9)	N4B—C2B	1.424 (10)
N4A—C7A	1.457 (10)	N4B—C7B	1.452 (10)
N4A—C4A	1.460 (10)	N4B—C4B	1.472 (9)
C1A—C2A	1.413 (11)	C1B—C2B	1.398 (11)
C1A—C8A	1.479 (11)	C1B—C18B	1.482 (11)
C2A—C3A	1.407 (11)	C2B—C3B	1.400 (11)
C3A—C18A	1.457 (11)	C3B—C8B	1.460 (11)
C4A—C5A	1.513 (11)	C4B—C5B	1.532 (10)
C4A—H4AA	0.99	C4B—H4BA	0.99
C4A—H4AB	0.99	C4B—H4BB	0.99
C5A—H5AA	0.99	C5B—H5BA	0.99
C5A—H5AB	0.99	C5B—H5BB	0.99
C6A—C7A	1.521 (11)	C6B—C7B	1.520 (10)
C6A—H6AA	0.99	C6B—H6BA	0.99
C6A—H6AB	0.99	C6B—H6BB	0.99
C7A—H7AA	0.99	C7B—H7BA	0.99
C7A—H7AB	0.99	C7B—H7BB	0.99
C8A—C12A	1.429 (11)	C8B—C12B	1.417 (11)
C8A—C9A	1.438 (11)	C8B—C9B	1.435 (11)
C9A—C10A	1.413 (12)	C9B—C10B	1.416 (12)
C9A—H9A	0.95	C9B—H9B	0.95
C10A—C11A	1.409 (12)	C10B—C11B	1.398 (12)
C10A—H10A	0.95	C10B—H10B	0.95
C11A—C12A	1.415 (12)	C11B—C12B	1.439 (12)
C11A—H11A	0.95	C11B—H11B	0.95
C12A—H12A	0.95	C12B—H12B	0.95
C13A—C14A	1.411 (12)	C13B—C17B	1.398 (13)
C13A—C17A	1.430 (11)	C13B—C14B	1.414 (12)
C13A—H13A	0.95	C13B—H13B	0.95
C14A—C15A	1.405 (13)	C14B—C15B	1.386 (13)
C14A—H14A	0.95	C14B—H14B	0.95
C15A—C16A	1.433 (12)	C15B—C16B	1.413 (13)
C15A—H15A	0.95	C15B—H15B	0.95
C16A—C17A	1.422 (12)	C16B—C17B	1.421 (13)
C16A—H16A	0.95	C16B—H16B	0.95
C17A—H17A	0.95	C17B—H17B	0.95
C18A—C22A	1.436 (11)	C18B—C19B	1.428 (11)
C18A—C19A	1.437 (11)	C18B—C22B	1.441 (10)
C19A—C20A	1.421 (11)	C19B—C20B	1.423 (12)
C19A—H19A	0.95	C19B—H19B	0.95
C20A—C21A	1.397 (11)	C20B—C21B	1.407 (13)
C20A—H20A	0.95	C20B—H20B	0.95
C21A—C22A	1.421 (12)	C21B—C22B	1.419 (12)
C21A—H21A	0.95	C21B—H21B	0.95
C22A—H22A	0.95	C22B—H22B	0.95
C23A—C27A	1.409 (12)	C23B—C27B	1.412 (12)
C23A—C24A	1.423 (12)	C23B—C24B	1.413 (13)
C23A—H23A	0.95	C23B—H23B	0.95
C24A—C25A	1.401 (12)	C24B—C25B	1.415 (16)
C24A—H24A	0.95	C24B—H24B	0.95
C25A—C26A	1.403 (12)	C25B—C26B	1.401 (15)
C25A—H25A	0.95	C25B—H25B	0.95
C26A—C27A	1.425 (13)	C26B—C27B	1.411 (12)
C26A—H26A	0.95	C26B—H26B	0.95
C27A—H27A	0.95	C27B—H27B	0.95
			
C12A—Fe1A—C15A	118.9 (4)	C9B—Fe1B—C15B	115.6 (4)
C12A—Fe1A—C9A	69.0 (3)	C9B—Fe1B—C10B	40.8 (3)
C15A—Fe1A—C9A	162.5 (4)	C15B—Fe1B—C10B	107.7 (4)
C12A—Fe1A—C14A	153.8 (3)	C9B—Fe1B—C14B	107.6 (3)
C15A—Fe1A—C14A	40.2 (4)	C15B—Fe1B—C14B	39.8 (4)
C9A—Fe1A—C14A	126.4 (4)	C10B—Fe1B—C14B	128.9 (4)
C12A—Fe1A—C17A	125.9 (3)	C9B—Fe1B—C13B	130.3 (4)
C15A—Fe1A—C17A	68.6 (4)	C15B—Fe1B—C13B	67.4 (4)
C9A—Fe1A—C17A	121.0 (4)	C10B—Fe1B—C13B	168.2 (4)
C14A—Fe1A—C17A	67.9 (4)	C14B—Fe1B—C13B	40.6 (3)
C12A—Fe1A—C16A	106.9 (4)	C9B—Fe1B—C11B	68.6 (4)
C15A—Fe1A—C16A	41.0 (4)	C15B—Fe1B—C11B	129.1 (4)
C9A—Fe1A—C16A	155.5 (4)	C10B—Fe1B—C11B	40.1 (3)
C14A—Fe1A—C16A	67.9 (3)	C14B—Fe1B—C11B	166.6 (4)
C17A—Fe1A—C16A	40.6 (4)	C13B—Fe1B—C11B	151.3 (4)
C12A—Fe1A—C11A	40.6 (3)	C9B—Fe1B—C12B	68.5 (3)
C15A—Fe1A—C11A	107.2 (3)	C15B—Fe1B—C12B	168.8 (4)
C9A—Fe1A—C11A	67.9 (4)	C10B—Fe1B—C12B	68.0 (3)
C14A—Fe1A—C11A	120.2 (4)	C14B—Fe1B—C12B	150.8 (3)
C17A—Fe1A—C11A	163.1 (3)	C13B—Fe1B—C12B	118.8 (3)
C16A—Fe1A—C11A	125.7 (4)	C11B—Fe1B—C12B	41.2 (3)
C12A—Fe1A—C8A	41.0 (3)	C9B—Fe1B—C16B	148.5 (4)
C15A—Fe1A—C8A	154.5 (3)	C15B—Fe1B—C16B	40.5 (4)
C9A—Fe1A—C8A	41.1 (3)	C10B—Fe1B—C16B	116.4 (4)
C14A—Fe1A—C8A	164.1 (3)	C14B—Fe1B—C16B	67.7 (4)
C17A—Fe1A—C8A	108.2 (3)	C13B—Fe1B—C16B	67.4 (4)
C16A—Fe1A—C8A	120.0 (3)	C11B—Fe1B—C16B	108.3 (4)
C11A—Fe1A—C8A	68.1 (3)	C12B—Fe1B—C16B	131.0 (4)
C12A—Fe1A—C10A	68.5 (3)	C9B—Fe1B—C8B	41.2 (3)
C15A—Fe1A—C10A	125.2 (4)	C15B—Fe1B—C8B	149.0 (4)
C9A—Fe1A—C10A	40.3 (3)	C10B—Fe1B—C8B	68.7 (3)
C14A—Fe1A—C10A	108.4 (4)	C14B—Fe1B—C8B	117.2 (3)
C17A—Fe1A—C10A	155.5 (4)	C13B—Fe1B—C8B	109.6 (3)
C16A—Fe1A—C10A	162.6 (4)	C11B—Fe1B—C8B	69.0 (3)
C11A—Fe1A—C10A	40.1 (3)	C12B—Fe1B—C8B	40.5 (3)
C8A—Fe1A—C10A	68.4 (3)	C16B—Fe1B—C8B	169.4 (4)
C12A—Fe1A—C13A	164.0 (3)	C9B—Fe1B—C17B	169.0 (3)
C15A—Fe1A—C13A	68.2 (4)	C15B—Fe1B—C17B	67.9 (4)
C9A—Fe1A—C13A	108.6 (4)	C10B—Fe1B—C17B	149.8 (4)
C14A—Fe1A—C13A	40.2 (3)	C14B—Fe1B—C17B	67.9 (3)
C17A—Fe1A—C13A	40.7 (3)	C13B—Fe1B—C17B	39.9 (4)
C16A—Fe1A—C13A	68.3 (4)	C11B—Fe1B—C17B	118.1 (4)
C11A—Fe1A—C13A	154.5 (3)	C12B—Fe1B—C17B	110.2 (3)
C8A—Fe1A—C13A	126.9 (3)	C16B—Fe1B—C17B	40.5 (4)
C10A—Fe1A—C13A	120.7 (3)	C8B—Fe1B—C17B	130.8 (3)
C22A—Fe2A—C27A	106.2 (4)	C19B—Fe2B—C24B	106.9 (4)
C22A—Fe2A—C20A	68.1 (3)	C19B—Fe2B—C25B	119.7 (4)
C27A—Fe2A—C20A	164.3 (4)	C24B—Fe2B—C25B	40.9 (4)
C22A—Fe2A—C26A	116.0 (4)	C19B—Fe2B—C20B	41.1 (3)
C27A—Fe2A—C26A	40.9 (4)	C24B—Fe2B—C20B	123.2 (4)
C20A—Fe2A—C26A	127.1 (4)	C25B—Fe2B—C20B	105.3 (4)
C22A—Fe2A—C19A	68.8 (3)	C19B—Fe2B—C23B	125.5 (4)
C27A—Fe2A—C19A	152.7 (4)	C24B—Fe2B—C23B	40.8 (4)
C20A—Fe2A—C19A	40.7 (3)	C25B—Fe2B—C23B	68.3 (4)
C26A—Fe2A—C19A	166.0 (4)	C20B—Fe2B—C23B	161.5 (4)
C22A—Fe2A—C21A	40.8 (3)	C19B—Fe2B—C21B	68.8 (4)
C27A—Fe2A—C21A	126.4 (4)	C24B—Fe2B—C21B	159.6 (4)
C20A—Fe2A—C21A	40.0 (3)	C25B—Fe2B—C21B	122.5 (4)
C26A—Fe2A—C21A	106.2 (3)	C20B—Fe2B—C21B	40.5 (4)
C19A—Fe2A—C21A	68.3 (3)	C23B—Fe2B—C21B	157.6 (4)
C22A—Fe2A—C23A	128.1 (4)	C19B—Fe2B—C27B	163.0 (3)
C27A—Fe2A—C23A	40.3 (3)	C24B—Fe2B—C27B	68.7 (4)
C20A—Fe2A—C23A	154.4 (4)	C25B—Fe2B—C27B	68.4 (4)
C26A—Fe2A—C23A	67.8 (4)	C20B—Fe2B—C27B	155.1 (4)
C19A—Fe2A—C23A	120.7 (3)	C23B—Fe2B—C27B	40.6 (3)
C21A—Fe2A—C23A	165.1 (4)	C21B—Fe2B—C27B	121.2 (4)
C22A—Fe2A—C18A	41.1 (3)	C19B—Fe2B—C22B	69.4 (3)
C27A—Fe2A—C18A	117.5 (3)	C24B—Fe2B—C22B	158.3 (4)
C20A—Fe2A—C18A	68.6 (3)	C25B—Fe2B—C22B	159.8 (4)
C26A—Fe2A—C18A	150.6 (4)	C20B—Fe2B—C22B	68.9 (4)
C19A—Fe2A—C18A	41.0 (3)	C23B—Fe2B—C22B	123.0 (4)
C21A—Fe2A—C18A	68.8 (3)	C21B—Fe2B—C22B	40.8 (3)
C23A—Fe2A—C18A	109.2 (3)	C27B—Fe2B—C22B	108.3 (4)
C22A—Fe2A—C24A	167.5 (4)	C19B—Fe2B—C26B	154.8 (4)
C27A—Fe2A—C24A	67.8 (3)	C24B—Fe2B—C26B	68.0 (5)
C20A—Fe2A—C24A	120.5 (3)	C25B—Fe2B—C26B	40.2 (4)
C26A—Fe2A—C24A	67.3 (3)	C20B—Fe2B—C26B	119.5 (4)
C19A—Fe2A—C24A	111.1 (3)	C23B—Fe2B—C26B	67.7 (4)
C21A—Fe2A—C24A	151.6 (4)	C21B—Fe2B—C26B	106.9 (4)
C23A—Fe2A—C24A	40.5 (3)	C27B—Fe2B—C26B	40.4 (3)
C18A—Fe2A—C24A	130.6 (3)	C22B—Fe2B—C26B	124.5 (4)
C22A—Fe2A—C25A	149.8 (4)	C19B—Fe2B—C18B	41.0 (3)
C27A—Fe2A—C25A	67.9 (4)	C24B—Fe2B—C18B	122.2 (4)
C20A—Fe2A—C25A	109.2 (3)	C25B—Fe2B—C18B	156.6 (4)
C26A—Fe2A—C25A	39.9 (3)	C20B—Fe2B—C18B	68.8 (3)
C19A—Fe2A—C25A	130.0 (3)	C23B—Fe2B—C18B	109.7 (4)
C21A—Fe2A—C25A	117.4 (3)	C21B—Fe2B—C18B	68.5 (3)
C23A—Fe2A—C25A	67.5 (4)	C27B—Fe2B—C18B	126.3 (4)
C18A—Fe2A—C25A	168.3 (3)	C22B—Fe2B—C18B	41.2 (3)
C24A—Fe2A—C25A	39.7 (3)	C26B—Fe2B—C18B	162.5 (4)
C6A—O1A—C5A	109.7 (7)	C6B—O1B—C5B	110.1 (6)
N2A—N1A—C3A	120.9 (7)	N2B—N1B—C3B	120.7 (7)
N1A—N2A—N3A	121.9 (7)	N1B—N2B—N3B	122.1 (7)
N2A—N3A—C1A	120.2 (7)	N2B—N3B—C1B	119.7 (7)
C2A—N4A—C7A	117.2 (6)	C2B—N4B—C7B	118.7 (6)
C2A—N4A—C4A	120.1 (6)	C2B—N4B—C4B	119.4 (6)
C7A—N4A—C4A	113.6 (6)	C7B—N4B—C4B	113.8 (6)
N3A—C1A—C2A	121.0 (7)	N3B—C1B—C2B	121.1 (7)
N3A—C1A—C8A	115.1 (7)	N3B—C1B—C18B	114.6 (7)
C2A—C1A—C8A	123.9 (7)	C2B—C1B—C18B	124.2 (7)
C3A—C2A—C1A	115.4 (7)	C1B—C2B—C3B	116.4 (7)
C3A—C2A—N4A	125.6 (7)	C1B—C2B—N4B	118.7 (7)
C1A—C2A—N4A	119.0 (7)	C3B—C2B—N4B	125.0 (7)
N1A—C3A—C2A	120.3 (7)	N1B—C3B—C2B	119.5 (8)
N1A—C3A—C18A	113.6 (7)	N1B—C3B—C8B	114.1 (7)
C2A—C3A—C18A	126.1 (7)	C2B—C3B—C8B	126.3 (7)
N4A—C4A—C5A	108.2 (7)	N4B—C4B—C5B	107.2 (6)
N4A—C4A—H4AA	110.1	N4B—C4B—H4BA	110.3
C5A—C4A—H4AA	110.1	C5B—C4B—H4BA	110.3
N4A—C4A—H4AB	110.1	N4B—C4B—H4BB	110.3
C5A—C4A—H4AB	110.1	C5B—C4B—H4BB	110.3
H4AA—C4A—H4AB	108.4	H4BA—C4B—H4BB	108.5
O1A—C5A—C4A	111.0 (7)	O1B—C5B—C4B	110.4 (6)
O1A—C5A—H5AA	109.4	O1B—C5B—H5BA	109.6
C4A—C5A—H5AA	109.4	C4B—C5B—H5BA	109.6
O1A—C5A—H5AB	109.4	O1B—C5B—H5BB	109.6
C4A—C5A—H5AB	109.4	C4B—C5B—H5BB	109.6
H5AA—C5A—H5AB	108	H5BA—C5B—H5BB	108.1
O1A—C6A—C7A	111.6 (7)	O1B—C6B—C7B	112.1 (6)
O1A—C6A—H6AA	109.3	O1B—C6B—H6BA	109.2
C7A—C6A—H6AA	109.3	C7B—C6B—H6BA	109.2
O1A—C6A—H6AB	109.3	O1B—C6B—H6BB	109.2
C7A—C6A—H6AB	109.3	C7B—C6B—H6BB	109.2
H6AA—C6A—H6AB	108	H6BA—C6B—H6BB	107.9
N4A—C7A—C6A	109.2 (7)	N4B—C7B—C6B	109.4 (7)
N4A—C7A—H7AA	109.8	N4B—C7B—H7BA	109.8
C6A—C7A—H7AA	109.8	C6B—C7B—H7BA	109.8
N4A—C7A—H7AB	109.8	N4B—C7B—H7BB	109.8
C6A—C7A—H7AB	109.8	C6B—C7B—H7BB	109.8
H7AA—C7A—H7AB	108.3	H7BA—C7B—H7BB	108.2
C12A—C8A—C9A	107.2 (7)	C12B—C8B—C9B	106.9 (7)
C12A—C8A—C1A	123.4 (7)	C12B—C8B—C3B	128.7 (7)
C9A—C8A—C1A	129.3 (7)	C9B—C8B—C3B	124.4 (7)
C12A—C8A—Fe1A	68.6 (5)	C12B—C8B—Fe1B	69.5 (4)
C9A—C8A—Fe1A	69.2 (5)	C9B—C8B—Fe1B	68.5 (4)
C1A—C8A—Fe1A	124.4 (6)	C3B—C8B—Fe1B	125.9 (5)
C10A—C9A—C8A	108.0 (8)	C10B—C9B—C8B	108.0 (7)
C10A—C9A—Fe1A	70.2 (5)	C10B—C9B—Fe1B	70.0 (5)
C8A—C9A—Fe1A	69.7 (5)	C8B—C9B—Fe1B	70.4 (4)
C10A—C9A—H9A	126	C10B—C9B—H9B	126
C8A—C9A—H9A	126	C8B—C9B—H9B	126
Fe1A—C9A—H9A	125.7	Fe1B—C9B—H9B	125.3
C11A—C10A—C9A	108.2 (8)	C11B—C10B—C9B	109.2 (7)
C11A—C10A—Fe1A	69.8 (5)	C11B—C10B—Fe1B	70.2 (5)
C9A—C10A—Fe1A	69.4 (5)	C9B—C10B—Fe1B	69.2 (4)
C11A—C10A—H10A	125.9	C11B—C10B—H10B	125.4
C9A—C10A—H10A	125.9	C9B—C10B—H10B	125.4
Fe1A—C10A—H10A	126.4	Fe1B—C10B—H10B	126.7
C10A—C11A—C12A	108.8 (7)	C10B—C11B—C12B	107.2 (8)
C10A—C11A—Fe1A	70.1 (5)	C10B—C11B—Fe1B	69.7 (5)
C12A—C11A—Fe1A	68.8 (5)	C12B—C11B—Fe1B	69.5 (4)
C10A—C11A—H11A	125.6	C10B—C11B—H11B	126.4
C12A—C11A—H11A	125.6	C12B—C11B—H11B	126.4
Fe1A—C11A—H11A	127.1	Fe1B—C11B—H11B	126
C11A—C12A—C8A	107.8 (7)	C8B—C12B—C11B	108.6 (7)
C11A—C12A—Fe1A	70.6 (5)	C8B—C12B—Fe1B	70.0 (4)
C8A—C12A—Fe1A	70.4 (5)	C11B—C12B—Fe1B	69.3 (4)
C11A—C12A—H12A	126.1	C8B—C12B—H12B	125.7
C8A—C12A—H12A	126.1	C11B—C12B—H12B	125.7
Fe1A—C12A—H12A	124.5	Fe1B—C12B—H12B	126.6
C14A—C13A—C17A	107.2 (8)	C17B—C13B—C14B	108.8 (8)
C14A—C13A—Fe1A	69.3 (5)	C17B—C13B—Fe1B	70.7 (5)
C17A—C13A—Fe1A	69.0 (5)	C14B—C13B—Fe1B	69.6 (5)
C14A—C13A—H13A	126.4	C17B—C13B—H13B	125.6
C17A—C13A—H13A	126.4	C14B—C13B—H13B	125.6
Fe1A—C13A—H13A	126.8	Fe1B—C13B—H13B	125.7
C15A—C14A—C13A	109.6 (8)	C15B—C14B—C13B	107.6 (8)
C15A—C14A—Fe1A	69.7 (5)	C15B—C14B—Fe1B	69.9 (5)
C13A—C14A—Fe1A	70.5 (5)	C13B—C14B—Fe1B	69.8 (5)
C15A—C14A—H14A	125.2	C15B—C14B—H14B	126.2
C13A—C14A—H14A	125.2	C13B—C14B—H14B	126.2
Fe1A—C14A—H14A	126.2	Fe1B—C14B—H14B	125.6
C14A—C15A—C16A	107.5 (8)	C14B—C15B—C16B	108.9 (8)
C14A—C15A—Fe1A	70.1 (5)	C14B—C15B—Fe1B	70.3 (5)
C16A—C15A—Fe1A	69.8 (5)	C16B—C15B—Fe1B	70.4 (5)
C14A—C15A—H15A	126.2	C14B—C15B—H15B	125.5
C16A—C15A—H15A	126.2	C16B—C15B—H15B	125.5
Fe1A—C15A—H15A	125.4	Fe1B—C15B—H15B	125.3
C17A—C16A—C15A	107.6 (8)	C15B—C16B—C17B	107.4 (8)
C17A—C16A—Fe1A	69.6 (5)	C15B—C16B—Fe1B	69.1 (5)
C15A—C16A—Fe1A	69.2 (5)	C17B—C16B—Fe1B	70.1 (5)
C17A—C16A—H16A	126.2	C15B—C16B—H16B	126.3
C15A—C16A—H16A	126.2	C17B—C16B—H16B	126.3
Fe1A—C16A—H16A	126.6	Fe1B—C16B—H16B	126.1
C16A—C17A—C13A	108.1 (8)	C13B—C17B—C16B	107.3 (8)
C16A—C17A—Fe1A	69.8 (5)	C13B—C17B—Fe1B	69.4 (5)
C13A—C17A—Fe1A	70.2 (5)	C16B—C17B—Fe1B	69.4 (5)
C16A—C17A—H17A	126	C13B—C17B—H17B	126.3
C13A—C17A—H17A	126	C16B—C17B—H17B	126.3
Fe1A—C17A—H17A	125.6	Fe1B—C17B—H17B	126.4
C22A—C18A—C19A	106.6 (7)	C19B—C18B—C22B	107.3 (7)
C22A—C18A—C3A	123.9 (7)	C19B—C18B—C1B	124.5 (7)
C19A—C18A—C3A	129.5 (7)	C22B—C18B—C1B	128.2 (7)
C22A—C18A—Fe2A	68.4 (4)	C19B—C18B—Fe2B	68.0 (4)
C19A—C18A—Fe2A	69.2 (4)	C22B—C18B—Fe2B	68.7 (5)
C3A—C18A—Fe2A	125.1 (5)	C1B—C18B—Fe2B	126.4 (6)
C20A—C19A—C18A	107.7 (7)	C20B—C19B—C18B	108.2 (7)
C20A—C19A—Fe2A	69.4 (5)	C20B—C19B—Fe2B	69.9 (5)
C18A—C19A—Fe2A	69.8 (4)	C18B—C19B—Fe2B	71.0 (5)
C20A—C19A—H19A	126.2	C20B—C19B—H19B	125.9
C18A—C19A—H19A	126.2	C18B—C19B—H19B	125.9
Fe2A—C19A—H19A	126.2	Fe2B—C19B—H19B	124.8
C21A—C20A—C19A	109.3 (7)	C21B—C20B—C19B	108.0 (8)
C21A—C20A—Fe2A	70.3 (5)	C21B—C20B—Fe2B	69.9 (5)
C19A—C20A—Fe2A	70.0 (5)	C19B—C20B—Fe2B	69.0 (5)
C21A—C20A—H20A	125.4	C21B—C20B—H20B	126
C19A—C20A—H20A	125.4	C19B—C20B—H20B	126
Fe2A—C20A—H20A	125.9	Fe2B—C20B—H20B	126.7
C20A—C21A—C22A	107.9 (7)	C20B—C21B—C22B	109.0 (8)
C20A—C21A—Fe2A	69.7 (5)	C20B—C21B—Fe2B	69.6 (5)
C22A—C21A—Fe2A	68.9 (5)	C22B—C21B—Fe2B	69.8 (5)
C20A—C21A—H21A	126.1	C20B—C21B—H21B	125.5
C22A—C21A—H21A	126.1	C22B—C21B—H21B	125.5
Fe2A—C21A—H21A	126.9	Fe2B—C21B—H21B	126.8
C21A—C22A—C18A	108.6 (7)	C21B—C22B—C18B	107.4 (7)
C21A—C22A—Fe2A	70.3 (5)	C21B—C22B—Fe2B	69.5 (5)
C18A—C22A—Fe2A	70.5 (5)	C18B—C22B—Fe2B	70.1 (5)
C21A—C22A—H22A	125.7	C21B—C22B—H22B	126.3
C18A—C22A—H22A	125.7	C18B—C22B—H22B	126.3
Fe2A—C22A—H22A	125.1	Fe2B—C22B—H22B	125.7
C27A—C23A—C24A	107.8 (8)	C27B—C23B—C24B	108.4 (9)
C27A—C23A—Fe2A	69.3 (5)	C27B—C23B—Fe2B	69.9 (5)
C24A—C23A—Fe2A	70.1 (5)	C24B—C23B—Fe2B	69.2 (5)
C27A—C23A—H23A	126.1	C27B—C23B—H23B	125.8
C24A—C23A—H23A	126.1	C24B—C23B—H23B	125.8
Fe2A—C23A—H23A	126.1	Fe2B—C23B—H23B	126.7
C25A—C24A—C23A	108.1 (8)	C23B—C24B—C25B	107.6 (10)
C25A—C24A—Fe2A	70.1 (5)	C23B—C24B—Fe2B	70.0 (5)
C23A—C24A—Fe2A	69.4 (5)	C25B—C24B—Fe2B	69.8 (6)
C25A—C24A—H24A	126	C23B—C24B—H24B	126.2
C23A—C24A—H24A	126	C25B—C24B—H24B	126.2
Fe2A—C24A—H24A	126	Fe2B—C24B—H24B	125.6
C24A—C25A—C26A	108.5 (8)	C26B—C25B—C24B	107.9 (9)
C24A—C25A—Fe2A	70.1 (5)	C26B—C25B—Fe2B	70.5 (6)
C26A—C25A—Fe2A	69.4 (5)	C24B—C25B—Fe2B	69.3 (6)
C24A—C25A—H25A	125.8	C26B—C25B—H25B	126.1
C26A—C25A—H25A	125.8	C24B—C25B—H25B	126.1
Fe2A—C25A—H25A	126.3	Fe2B—C25B—H25B	125.7
C25A—C26A—C27A	108.1 (8)	C25B—C26B—C27B	108.9 (9)
C25A—C26A—Fe2A	70.7 (5)	C25B—C26B—Fe2B	69.3 (6)
C27A—C26A—Fe2A	69.3 (5)	C27B—C26B—Fe2B	69.5 (5)
C25A—C26A—H26A	126	C25B—C26B—H26B	125.6
C27A—C26A—H26A	126	C27B—C26B—H26B	125.6
Fe2A—C26A—H26A	125.6	Fe2B—C26B—H26B	127.2
C23A—C27A—C26A	107.6 (8)	C26B—C27B—C23B	107.2 (9)
C23A—C27A—Fe2A	70.4 (5)	C26B—C27B—Fe2B	70.1 (5)
C26A—C27A—Fe2A	69.8 (5)	C23B—C27B—Fe2B	69.5 (5)
C23A—C27A—H27A	126.2	C26B—C27B—H27B	126.4
C26A—C27A—H27A	126.2	C23B—C27B—H27B	126.4
Fe2A—C27A—H27A	125.1	Fe2B—C27B—H27B	125.6
			
C3A—N1A—N2A—N3A	−0.1 (12)	C3B—N1B—N2B—N3B	−0.2 (12)
N1A—N2A—N3A—C1A	4.5 (12)	N1B—N2B—N3B—C1B	−4.4 (12)
N2A—N3A—C1A—C2A	−3.5 (12)	N2B—N3B—C1B—C2B	2.2 (12)
N2A—N3A—C1A—C8A	176.6 (7)	N2B—N3B—C1B—C18B	−177.9 (7)
N3A—C1A—C2A—C3A	−1.6 (11)	N3B—C1B—C2B—C3B	4.1 (12)
C8A—C1A—C2A—C3A	178.3 (7)	C18B—C1B—C2B—C3B	−175.8 (8)
N3A—C1A—C2A—N4A	177.2 (7)	N3B—C1B—C2B—N4B	−175.7 (7)
C8A—C1A—C2A—N4A	−3.0 (12)	C18B—C1B—C2B—N4B	4.5 (12)
C7A—N4A—C2A—C3A	−101.9 (9)	C7B—N4B—C2B—C1B	−78.2 (9)
C4A—N4A—C2A—C3A	43.0 (11)	C4B—N4B—C2B—C1B	134.8 (8)
C7A—N4A—C2A—C1A	79.5 (9)	C7B—N4B—C2B—C3B	102.1 (9)
C4A—N4A—C2A—C1A	−135.6 (8)	C4B—N4B—C2B—C3B	−44.9 (11)
N2A—N1A—C3A—C2A	−5.2 (12)	N2B—N1B—C3B—C2B	6.8 (12)
N2A—N1A—C3A—C18A	175.8 (7)	N2B—N1B—C3B—C8B	−177.5 (7)
C1A—C2A—C3A—N1A	5.8 (11)	C1B—C2B—C3B—N1B	−8.4 (12)
N4A—C2A—C3A—N1A	−172.9 (7)	N4B—C2B—C3B—N1B	171.3 (7)
C1A—C2A—C3A—C18A	−175.3 (7)	C1B—C2B—C3B—C8B	176.4 (8)
N4A—C2A—C3A—C18A	6.0 (13)	N4B—C2B—C3B—C8B	−3.9 (13)
C2A—N4A—C4A—C5A	159.5 (7)	C2B—N4B—C4B—C5B	−155.5 (7)
C7A—N4A—C4A—C5A	−54.4 (9)	C7B—N4B—C4B—C5B	55.9 (8)
C6A—O1A—C5A—C4A	−62.3 (9)	C6B—O1B—C5B—C4B	62.1 (8)
N4A—C4A—C5A—O1A	58.1 (9)	N4B—C4B—C5B—O1B	−59.2 (8)
C5A—O1A—C6A—C7A	60.3 (10)	C5B—O1B—C6B—C7B	−59.0 (9)
C2A—N4A—C7A—C6A	−160.1 (7)	C2B—N4B—C7B—C6B	157.9 (7)
C4A—N4A—C7A—C6A	52.7 (9)	C4B—N4B—C7B—C6B	−53.3 (8)
O1A—C6A—C7A—N4A	−54.9 (10)	O1B—C6B—C7B—N4B	53.5 (9)
N3A—C1A—C8A—C12A	39.9 (11)	N1B—C3B—C8B—C12B	143.5 (8)
C2A—C1A—C8A—C12A	−139.9 (8)	C2B—C3B—C8B—C12B	−41.1 (13)
N3A—C1A—C8A—C9A	−135.8 (9)	N1B—C3B—C8B—C9B	−34.9 (11)
C2A—C1A—C8A—C9A	44.3 (13)	C2B—C3B—C8B—C9B	140.5 (8)
N3A—C1A—C8A—Fe1A	−45.5 (10)	N1B—C3B—C8B—Fe1B	52.0 (10)
C2A—C1A—C8A—Fe1A	134.6 (7)	C2B—C3B—C8B—Fe1B	−132.6 (8)
C12A—C8A—C9A—C10A	1.8 (10)	C12B—C8B—C9B—C10B	1.0 (8)
C1A—C8A—C9A—C10A	178.1 (8)	C3B—C8B—C9B—C10B	179.7 (7)
Fe1A—C8A—C9A—C10A	60.0 (6)	Fe1B—C8B—C9B—C10B	60.1 (5)
C12A—C8A—C9A—Fe1A	−58.2 (6)	C12B—C8B—C9B—Fe1B	−59.1 (5)
C1A—C8A—C9A—Fe1A	118.1 (9)	C3B—C8B—C9B—Fe1B	119.6 (8)
C8A—C9A—C10A—C11A	−0.5 (10)	C8B—C9B—C10B—C11B	−1.4 (9)
Fe1A—C9A—C10A—C11A	59.2 (6)	Fe1B—C9B—C10B—C11B	59.0 (6)
C8A—C9A—C10A—Fe1A	−59.7 (6)	C8B—C9B—C10B—Fe1B	−60.4 (5)
C9A—C10A—C11A—C12A	−1.0 (10)	C9B—C10B—C11B—C12B	1.2 (9)
Fe1A—C10A—C11A—C12A	58.0 (6)	Fe1B—C10B—C11B—C12B	59.6 (5)
C9A—C10A—C11A—Fe1A	−59.0 (6)	C9B—C10B—C11B—Fe1B	−58.4 (6)
C10A—C11A—C12A—C8A	2.2 (10)	C9B—C8B—C12B—C11B	−0.2 (8)
Fe1A—C11A—C12A—C8A	60.9 (6)	C3B—C8B—C12B—C11B	−178.9 (8)
C10A—C11A—C12A—Fe1A	−58.8 (6)	Fe1B—C8B—C12B—C11B	−58.7 (5)
C9A—C8A—C12A—C11A	−2.4 (10)	C9B—C8B—C12B—Fe1B	58.5 (5)
C1A—C8A—C12A—C11A	−179.0 (7)	C3B—C8B—C12B—Fe1B	−120.2 (8)
Fe1A—C8A—C12A—C11A	−61.1 (6)	C10B—C11B—C12B—C8B	−0.6 (9)
C9A—C8A—C12A—Fe1A	58.6 (6)	Fe1B—C11B—C12B—C8B	59.1 (5)
C1A—C8A—C12A—Fe1A	−117.9 (8)	C10B—C11B—C12B—Fe1B	−59.7 (6)
C17A—C13A—C14A—C15A	0.0 (10)	C17B—C13B—C14B—C15B	0.1 (9)
Fe1A—C13A—C14A—C15A	−58.9 (6)	Fe1B—C13B—C14B—C15B	−60.0 (6)
C17A—C13A—C14A—Fe1A	58.9 (6)	C17B—C13B—C14B—Fe1B	60.0 (6)
C13A—C14A—C15A—C16A	−0.7 (10)	C13B—C14B—C15B—C16B	−0.1 (10)
Fe1A—C14A—C15A—C16A	−60.1 (6)	Fe1B—C14B—C15B—C16B	−60.0 (6)
C13A—C14A—C15A—Fe1A	59.4 (6)	C13B—C14B—C15B—Fe1B	59.9 (6)
C14A—C15A—C16A—C17A	1.1 (10)	C14B—C15B—C16B—C17B	0.1 (10)
Fe1A—C15A—C16A—C17A	−59.2 (6)	Fe1B—C15B—C16B—C17B	−59.9 (6)
C14A—C15A—C16A—Fe1A	60.3 (6)	C14B—C15B—C16B—Fe1B	60.0 (6)
C15A—C16A—C17A—C13A	−1.2 (10)	C14B—C13B—C17B—C16B	0.0 (9)
Fe1A—C16A—C17A—C13A	−60.0 (6)	Fe1B—C13B—C17B—C16B	59.3 (6)
C15A—C16A—C17A—Fe1A	58.9 (6)	C14B—C13B—C17B—Fe1B	−59.3 (6)
C14A—C13A—C17A—C16A	0.7 (9)	C15B—C16B—C17B—C13B	0.0 (9)
Fe1A—C13A—C17A—C16A	59.8 (6)	Fe1B—C16B—C17B—C13B	−59.3 (6)
C14A—C13A—C17A—Fe1A	−59.0 (6)	C15B—C16B—C17B—Fe1B	59.3 (6)
N1A—C3A—C18A—C22A	37.8 (11)	N3B—C1B—C18B—C19B	−37.1 (11)
C2A—C3A—C18A—C22A	−141.1 (8)	C2B—C1B—C18B—C19B	142.7 (8)
N1A—C3A—C18A—C19A	−139.2 (8)	N3B—C1B—C18B—C22B	139.9 (9)
C2A—C3A—C18A—C19A	41.9 (13)	C2B—C1B—C18B—C22B	−40.3 (13)
N1A—C3A—C18A—Fe2A	−48.2 (10)	N3B—C1B—C18B—Fe2B	49.5 (10)
C2A—C3A—C18A—Fe2A	132.8 (7)	C2B—C1B—C18B—Fe2B	−130.6 (7)
C22A—C18A—C19A—C20A	0.8 (9)	C22B—C18B—C19B—C20B	2.7 (9)
C3A—C18A—C19A—C20A	178.2 (8)	C1B—C18B—C19B—C20B	−179.8 (7)
Fe2A—C18A—C19A—C20A	59.3 (5)	Fe2B—C18B—C19B—C20B	60.3 (6)
C22A—C18A—C19A—Fe2A	−58.4 (5)	C22B—C18B—C19B—Fe2B	−57.6 (6)
C3A—C18A—C19A—Fe2A	118.9 (8)	C1B—C18B—C19B—Fe2B	120.0 (8)
C18A—C19A—C20A—C21A	0.0 (9)	C18B—C19B—C20B—C21B	−1.7 (9)
Fe2A—C19A—C20A—C21A	59.5 (6)	Fe2B—C19B—C20B—C21B	59.2 (6)
C18A—C19A—C20A—Fe2A	−59.6 (5)	C18B—C19B—C20B—Fe2B	−61.0 (6)
C19A—C20A—C21A—C22A	−0.8 (9)	C19B—C20B—C21B—C22B	0.1 (10)
Fe2A—C20A—C21A—C22A	58.5 (5)	Fe2B—C20B—C21B—C22B	58.7 (6)
C19A—C20A—C21A—Fe2A	−59.3 (6)	C19B—C20B—C21B—Fe2B	−58.6 (6)
C20A—C21A—C22A—C18A	1.3 (9)	C20B—C21B—C22B—C18B	1.6 (10)
Fe2A—C21A—C22A—C18A	60.3 (5)	Fe2B—C21B—C22B—C18B	60.2 (6)
C20A—C21A—C22A—Fe2A	−59.0 (6)	C20B—C21B—C22B—Fe2B	−58.6 (6)
C19A—C18A—C22A—C21A	−1.3 (9)	C19B—C18B—C22B—C21B	−2.6 (9)
C3A—C18A—C22A—C21A	−178.9 (7)	C1B—C18B—C22B—C21B	180.0 (8)
Fe2A—C18A—C22A—C21A	−60.3 (5)	Fe2B—C18B—C22B—C21B	−59.7 (6)
C19A—C18A—C22A—Fe2A	58.9 (5)	C19B—C18B—C22B—Fe2B	57.2 (6)
C3A—C18A—C22A—Fe2A	−118.6 (7)	C1B—C18B—C22B—Fe2B	−120.3 (9)
C27A—C23A—C24A—C25A	−0.5 (9)	C27B—C23B—C24B—C25B	−0.8 (10)
Fe2A—C23A—C24A—C25A	−59.7 (6)	Fe2B—C23B—C24B—C25B	−59.9 (7)
C27A—C23A—C24A—Fe2A	59.3 (5)	C27B—C23B—C24B—Fe2B	59.1 (6)
C23A—C24A—C25A—C26A	0.3 (9)	C23B—C24B—C25B—C26B	−0.2 (11)
Fe2A—C24A—C25A—C26A	−59.0 (6)	Fe2B—C24B—C25B—C26B	−60.3 (7)
C23A—C24A—C25A—Fe2A	59.3 (6)	C23B—C24B—C25B—Fe2B	60.1 (6)
C24A—C25A—C26A—C27A	0.0 (9)	C24B—C25B—C26B—C27B	1.1 (11)
Fe2A—C25A—C26A—C27A	−59.5 (6)	Fe2B—C25B—C26B—C27B	−58.4 (7)
C24A—C25A—C26A—Fe2A	59.4 (6)	C24B—C25B—C26B—Fe2B	59.5 (7)
C24A—C23A—C27A—C26A	0.4 (9)	C25B—C26B—C27B—C23B	−1.6 (10)
Fe2A—C23A—C27A—C26A	60.2 (6)	Fe2B—C26B—C27B—C23B	−59.8 (6)
C24A—C23A—C27A—Fe2A	−59.8 (5)	C25B—C26B—C27B—Fe2B	58.2 (7)
C25A—C26A—C27A—C23A	−0.3 (9)	C24B—C23B—C27B—C26B	1.5 (10)
Fe2A—C26A—C27A—C23A	−60.6 (6)	Fe2B—C23B—C27B—C26B	60.2 (6)
C25A—C26A—C27A—Fe2A	60.3 (6)	C24B—C23B—C27B—Fe2B	−58.7 (6)

### Hydrogen-bond geometry (Å, º)

**Table:**

*D*—H···*A*	*D*—H	H···*A*	*D*···*A*	*D*—H···*A*
C21*B*—H21*B*···N2*A*^i^	0.95	2.56	3.300 (12)	135
C21*B*—H21*B*···N3*A*^i^	0.95	2.52	3.415 (10)	156
C24*A*—H24*A*···O1*B*	0.95	2.51	3.401 (10)	156

Symmetry code: (i) −*x*+1/2, *y*+1/2, *z*+1/2.
